# Reference values for glomerular filtration rate in healthy Brazilian adults

**DOI:** 10.1186/1471-2369-14-54

**Published:** 2013-02-28

**Authors:** Ariana Aguiar Soares, Aline Bodanese Prates, Letícia Schwerz Weinert, Francisco Veríssimo Veronese, Mirela Jobim de Azevedo, Sandra Pinho Silveiro

**Affiliations:** 1 Postgraduate Program in Medical Sciences: Endocrinology, Universidade Federal do Rio Grande do Sul (UFRGS), Porto Alegre, RS 90040-060, Brazil; 2 Nephrology Division, Hospital de Clínicas de Porto Alegre (HCPA), Porto Alegre, RS 90035-903, Brazil; 3 Endocrinology Division, HCPA, Rua Ramiro Barcelos, 2350 – Prédio 12, 4º andar Porto Alegre, RS, Brazil

**Keywords:** Aging, ^51^Cr-EDTA, Gender, Glomerular filtration rate, Reference values, Renal function

## Abstract

**Background:**

Glomerular filtration rate (GFR) is the best index of renal function, but age, gender and ethnicity can putatively affect its values. The aim of this study was to establish reference values for GFR in healthy Brazilian subjects while taking these factors into account.

**Methods:**

In this cross-sectional study, GFR was measured by the ^51^Cr-EDTA single-injection method. GFR reference values were developed according to CLSI Guidelines for Defining, Establishing, and Verifying Reference Intervals in the Clinical Laboratory (CLSI C28 protocol).

**Results:**

The age range of the 285 healthy individuals was 19 to 70 years, 57% were females, and GFR was 106 ± 18 mL/min/1.73 m^2^. There was no difference between male and female GFRs (108 ± 18 vs. 104 ± 18 mL/min/1.73 m^2^ respectively, P = 0.134), and reference values were therefore developed from the pooled sample. GFR values were lower in subjects aged ≥45 years as compared with those younger than 45 years (98 ± 15 vs.112 ± 18 mL/min/1.73 m^2^, P < 0.001). Based on mean ± 2 SD, GFR reference values were 76 to 148 mL/min/1.73 m^2^ for subjects younger than 45 years and 68 to 128 mL/min/1.73 m^2^ for individuals older than 45 years, irrespective of gender.

**Conclusion:**

The age-adjusted reference intervals reported may be reliably adopted to evaluate kidney function, since they are based on recommended standards.

## Background

The National Kidney Foundation (NKF) recommends estimating glomerular filtration rate (GFR) with creatinine-based equations, such as the Modification of Diet in Renal Disease (MDRD) study equation or the recently developed, and more accurate, Chronic Kidney Disease Epidemiology (CKD-EPI) collaboration group equation (http://www.kidney.org, retrieved January 2013) [1-4]. However, some clinical situations require a more precise evaluation of GFR, such as before kidney transplantation, extremes of age and body size, severe malnutrition or obesity, diseases of the skeletal muscle, paraplegia or quadriplegia, vegetarian diet, and before administration of prolonged courses of toxic medications [5,6]. In this scenario, GFR should ideally be measured by conventional clearance methods, such as inulin, iohexol, iothalamate, or ^51^Cr-EDTA clearance.

GFR decline with aging is a well-known phenomenon, and reference values should take it into account [7,8]. However, some studies have suggested that GFR may also be affected by sex, with women exhibiting either higher [9], lower [7] or similar [10] GFR values as compared to men. An even more controversial issue, which remains unsettled, is the potential influence of ethnicity on GFR. Therefore, the aim of this study was to establish reference range values for GFR in healthy Brazilian individuals while taking these factors into account.

## Methods

### Study participants

This cross-sectional study was conducted in healthy Southern Brazilian volunteers recruited from the community and hospital staff. Two hundred and eighty-five volunteers (aged 19–70 years) were included, and their health status was checked by a medical interview, a complete physical examination, and basic laboratory tests (fasting plasma glucose, lipids, liver function tests and urinalysis). Exclusion criteria were the presence of kidney disease (as detected by urinary albumin excretion >30 mg/g creatinine in a spot urine sample and sediment analysis), impaired glucose tolerance (fasting plasma glucose >100 mg/dL), arterial hypertension (blood pressure levels higher than 140/90 mmHg), cardiovascular disease (history and previous medical records) or presence of any other active disease, body mass index (BMI) >35 kg/m^2^, history of cancer in the last 5 years and use of drugs (except for oral contraceptives and thyroid hormones).

Subjects were classified as white, black, or other as self-reported, pursuant to current recommendations. The body weight and height of subjects (not wearing shoes or coats) were measured using an anthropometric scale, with measurements recorded to the nearest 100 g for weight and the nearest 0.1 cm for height. BMI was calculated using the formula kg/m^2^. Blood pressure was measured in the sitting position after a 5-min rest.

### Laboratory measurements

#### Procedure for GFR measurement

GFR was measured by the ^51^Cr-EDTA single-injection method, and the procedure is described below [11]. 1) Patient preparation: caffeine drinks and exercise were avoided. A low-protein meal and adequate hydration during the test were prescribed. Body surface area (BSA) was calculated according to the Gehan and George formula: 0.0235 × ([100 × height]^0.42246^) × (weight^0.51456^) [12]. 2) Syringe preparation: a 5.55 MBq dose of ^51^Cr-EDTA was measured by volume, and the syringe was filled completely up to the tip of the needle. 3) Injection technique: the entire dose was injected into the bloodstream without extravasation. 4) Blood sampling: samples were collected from the contralateral arm, 2, 3 and 4 h post-injection. The specimens were centrifuged at 1000 *g* for 10 min and 2 mL of plasma were pipetted into counting tubes in duplicate. 5) Preparation of the standard: a predetermined volume of tracer (2 mL) was drawn up using a pipette, and the activity was emptied into the volumetric flask filled with water to the mark. 6) Counting: plasma samples were counted using appropriate standards and blanks for background in a well counter. 7) GFR calculation: the logarithm of the plasma activity was plotted as a function of time and the apparent zero-time plasma activity determined by extrapolation of the linear part of the curve. A constant correction factor of 0.87 was used for the missing AUC due to the fast exponential; therefore, the GFR was calculated as volume of distribution x 0.693 x 0.87 × 1000/t_1/2_ (according to Chantler), and expressed as mL/min/1.73 m^2^[13]. The mean intra-individual coefficient of variation of GFR at our laboratory is 12% [7].

Fasting plasma glucose was measured by the glucose oxidase method, and albuminuria, by immunoturbidimetry.

### Statistical analysis

Results were expressed as mean ± standard deviation or median (range or interquartile range), unless otherwise stated. The Kolmogorov–Smirnov (K-S) test was employed to assess the distribution of variables, and reference values were defined as mean ± 2 standard deviations. The minimum number of subjects to be included in each partition was 120, based on the recommendations of Clinical and Laboratory Standards Institute (CLSI) C28 protocol for determining reference intervals [14]. Reed’s criterion was used to identify outliers, as recommended by Fraser [15], as were stem-and-leaf plots and histograms. Correlation between variables was evaluated by Pearson’s correlation test. The unpaired Student’s *t*-test and chi-square tests were used as appropriate. Simple and multivariate regression analyses were employed to evaluate the influence of age and gender on GFR (dependent variable). The statistical package used was PASW Statistics 19, and the level of significance adopted was 5%.

### Ethics

The Hospital de Clínicas de Porto Alegre Research Ethics Committee approved the study protocol, and all subjects provided written informed consent.

## Results

Two hundred and eighty-five healthy adults, 162 (57%) women, 266 (93%) white, 19 (7%) black, aged 41 ± 13 years (range, 19–70 years), BMI 25 ± 3 kg/m^2^, were evaluated (Table 1). Overall, the GFR measured by ^51^Cr-EDTA clearance (^51^Cr-GFR) was 106 ± 18 mL/min/1.73 m^2^ (range, 67 to 153 mL/min/1.73 m^2^), with a Gaussian distribution.

**Table 1:** Clinical characteristics of the sample (285 healthy volunteers)

	**Overall sample (n = 285)**
Age (years)	41 ± 13
Skin color (white/black)*	266/19
Body mass index (kg/m^2^)	25 ± 3 (16–34)
Body surface area (m^2^)	1.80 ± 0.19 (1.32-2.33)

Data expressed as mean ± SD (range) or number of cases.

*Self-reported.

**Figure 1:**
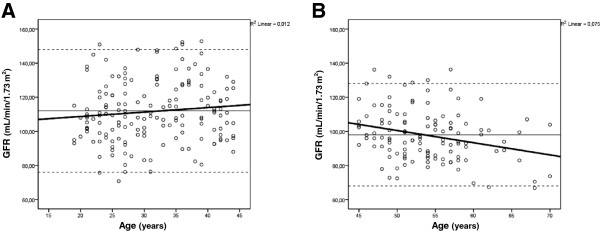
**Glomerular filtration rate (GFR) values presented as mean (continuous line) ± 2SD (dashed lines) and GFR regression equation (bold line) by age range.** Panel **A**: ages 20–45 years, regression: GFR = 103.4 + 0.26 x age; Panel **B**: ages 45–70 years, regression: GFR = 136.4 – 0.72 x age.

**Figure 2:**
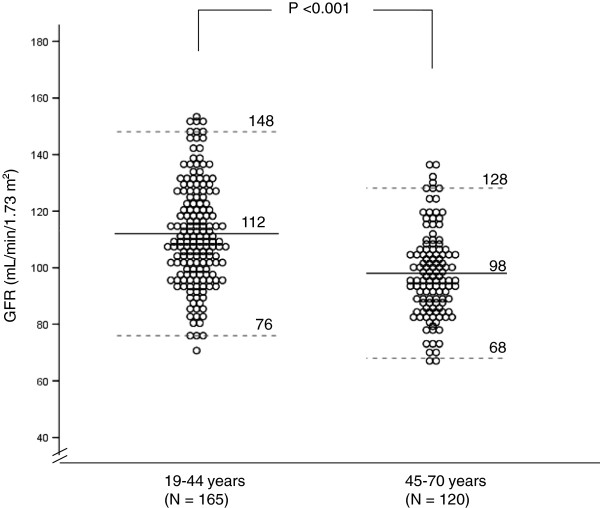
Individual glomerular filtration rate (GFR) values, expressed as mean ± 2SD (dashed lines), in healthy individuals aged <45 years vs. ≥45 years.

There was a significant inverse correlation between age and GFR (r = −0.33, P <0.001). Simple linear regression analysis confirmed the influence of age on GFR (r^2^ = 0.11, P <0.001). After stratifying data by age decades, we found that GFR starts to decline significantly after 45 years of age (Figure 1). We compared the slope of the GFR regression equation (y = a + bx) in subjects younger than 45 (GFR = 103.4 + 0.26 x age) vs. older than 45 years of age (GFR = 136.4 – 0.72 x age). The slope of younger subjects was not different from zero (one sample t test, P = 0.169), whereas the slope of the older group was consistent with a significant decrease in GFR (P = 0.003). Reference GFR values (mean ± 2 SD) were calculated, ranging from 76 to 148 mL/min/1.73 m^2^ for individuals aged <45 years and 68 to 128 mL/min/1.73 m^2^ for subjects older than 45 years. These values are depicted in Figure 2.

To assess the potential relation between GFR and gender, the 123 male subjects were compared to the 162 female participants. Men and women were matched by age (40 ± 14 vs. 41 ± 13 years, P = 0.66); BMI (25 ± 3 vs. 25 ± 4 kg/m^2^, P = 0.13), and ethnicity (93% vs. 94% white, P = 0.81), respectively. As expected, BSA was greater in men (1.9 ± 0.16 vs. 1.7 ± 0.15 m^2^, P < 0.001). After adjusting for BSA, the mean GFR was similar in men and women (108 ± 18 vs.104 ± 18 mL/min/1.73 m^2^ respectively, P = 0.13). The inverse correlation observed between GFR and age was similar for men and women (r = −0.45 and −0.33 respectively, P = 0.243). Simple linear regression analysis confirmed that gender did not influence GFR (r^2^ = −0.002; P = 0.47). Accordingly, multiple linear regression was performed with GFR as the dependent variable, and age and gender simultaneously as independent variables, yielding an r^2^ of 0.15, P < 0.001 for age only. On comparison between the 93 women and 72 men aged <45 years, there was no significant difference between GFRs (111 ± 18 vs. 113 ± 18 respectively, P = 0.51). Comparison between the 69 women and 51 men aged >45 years, however, showed a trend toward lower GFRs in the female group (96 ± 14 vs. 101 ± 16, P = 0.08).

To analyze the influence of skin color on GFR, we compared the 19 black individuals with the 266 white subjects. These groups were similar regarding age (38 ± 10 vs 41 ± 14 years respectively, P = 0.375), but GFR was higher in the black subjects (116 ± 19 vs 105 ± 18 mL/min/1.73 m^2^, P = 0.01), as was BMI (28 ± 3 vs 24 ± 3 kg/m^2^, P < 0.001). However, final GFR results were similar when analyzed with (106 mL/min/1.73 m²) or without (105 mL/min/1.73 m²) the black subjects.

The influence of BMI on GFR was also evaluated. Twenty-one of the 285 subjects had a BMI ≥30 kg/m^2^. The adjusted BSA-GFR was similar in obese and non-obese subjects (106±18 vs. 106±18 mL/min/1.73 m^2^ respectively, P = 0.951). Conversely, non-adjusted GFR was significantly higher in obese subjects (123±24 vs. 109±22 mL/min respectively, P = 0.006). Likewise, there was a significant positive correlation between BMI and unadjusted-BSA GFR (r = 0.29, P <0.001), but no correlation between BMI and adjusted GFR (r = −0.05, P = 0.359).

## Discussion

The present study confirms that age must be taken into account when establishing GFR reference values, since a decline in kidney function with age is a well-known phenomenon [10,16-18]. For the 20-to-45-year age group, GFR (mean ± 2 SD) ranged from 76 to 148 mL/min/1.73 m^2^, without distinction between men and women, versus 68 to 128 mL/min/1.73 m^2^ in subjects older than 45 years, with a trend toward lower GFRs in older women.

Compared to previous studies, our data showed slightly higher GFRs for younger individuals, probably because the participants of these studies were potential kidney donors [8-10,16,17], whereas the volunteers in our study were picked from a healthy population. This approach tends to minimize selection bias, since donors are mostly relatives of kidney recipients and may have a greater proportion of unrecognized renal disease [17]. Therefore, the results of the current study, which used a sample composed entirely of healthy individuals, are suitable to provide reference values.

Studies conducted several decades ago have clearly established that GFR declines with ageing. However, the vast majority of these studies were carried out on kidney donors. The seminal studies from the 1950s demonstrated that GFR decreases linearly with aging, with an increase in the rate of decline after the age of 50 years [10,18]. Granerus and Aurell [10] compiled the datasets of eight studies from the 1950s to 1980s and described a GFR decline of up to 4 mL/min/decade for ages <50 years and of 10 mL/min/decade for ages above this cutoff value. This pattern was confirmed by Slack and Wilson [19]. More recently, Poggio et al. [9] found that GFR declines at a rate of approximately 4 mL/min/decade in subjects younger than 45 years of age, similar to what has been previously reported. Grewal and Blake [16] report that GFR remains constant until the age of 40 years and then starts to decline; this is consistent with our results, which showed no GFR decline before the age of 45 years but a noticeable decrease in older subjects. This decrease is related to the normal physiological process of organ senescence and is associated with structural changes in the kidneys [20]. The mean number of nephrons in adults is around 900,000, ranging from 200,000 to 2,000,000 [21]. Studies of living kidney donors have shown that older donors exhibit a 30-45% reduction in the number of functioning glomeruli and a significantly lower GFR before donation as compared with their younger counterparts [22]. A remarkable study evaluated the renal function of 1203 living kidney donors and demonstrated that, although up to 70% of subjects over the age of 70 years had nephrosclerosis, the decline in kidney function with aging was not fully explained by this finding [8]. Further studies are still required to assess whether these age-related histologic abnormalities are predictive of adverse outcomes.

In our study, we found no significant difference in GFR values between men and women. Some studies corroborate this similarity [8,10,16,17]. A recent study reported higher GFR values for men, but the difference did not hold when GFR was normalized for BSA [8]. This is consistent with our findings, as we reported ^51^Cr-GFR corrected for BSA and no gender differences in GFR were demonstrated. A previous analysis of a small sample of healthy subjects described GFR values around 8% higher in men than in women, especially after the age of 30 years, but this difference disappeared when each age range was analyzed individually [7]. Only one study reported higher GFR values in women (3% higher as compared with men), but the authors maintain that this difference is unlikely to be clinically relevant [9]. Therefore, the vast majority of the studies reveals no difference between male and female GFR reference values. Our results suggest a trend toward lower GFRs in older women. A very recent study of 1878 healthy potential donors described interesting new findings, such as higher GFR/ECV (extracellular volume) values in women, reinforcing the notion that GFR should be corrected for ECV [23]. Expressing renal function as the GFR/ECV (mL/min/L) has a direct physiological interpretation, and can be an important adjunct to the usual adjustment of GFR for BSA [24].

Another aspect that has been questioned is the rate of age-related decline in renal function in men and women. Some authors have described a faster rate of GFR decrease in males [25]. Berg, analyzing a group of young (age 20–50 years) Swedish potential kidney donors, found a significant GFR decline only in men, ascribing a protective role to estrogens in pre-menopausal women [26], but this seems to be an isolated finding. In contrast, Ma et al. [27] reported a more marked decline in GFR values with age in Chinese women, but this also seems to constitute an atypical phenomenon. The majority of studies have failed to confirm any gender differences in age-related GFR decline [10,16,28], which is consistent with our findings.

The influence of ethnicity on GFR values has been assessed by some research groups. A Chinese study described lower GFR reference values [27] as compared with those of healthy Western populations [17], but higher than those reported for Indian adults [29]. These differences might be explained by peculiarities in dietary intake among distinct populations, by differences in GFR measurement methods, or, perhaps, by ethnicity itself. Therefore, due to this possible variation, the development of reference values of GFR for each specific population is important. To the best of our knowledge, ours is the first paper to report guideline-based GFR reference values for Brazilian individuals. It bears mentioning that the Brazilian population itself is composed of a massive “mix” of different ethnic groups. Indeed, subjects who declare themselves “white” actually represent different degrees of miscegenation between several black and white ethnicities, and the same applies to the subjects who report their skin color as “black”. Therefore, we chose not to stratify subjects by skin color for final analysis, since GFR values were not different when the black subjects were removed from the sample. Even though GFR was higher among black subjects, body weight was also greater in this group, which could represent a confounding factor, as obesity has been associated with hyperfiltration [30]. Furthermore, the number of self-reported black subjects was very small (n = 19). The potential role of ethnicity in GFR values still warrants further investigation.

Regarding the influence of elevated BMI on GFR, we found that the crude GFR (unadjusted for BSA) was significantly higher in obese subjects. As the current NKDEP recommendation is that measured GFR should be adjusted for BSA (to address symmorphosis in kidney function), we chose to merge the final results of obese and non-obese subjects, but also presented the results of each group of BMI separately [31]. It has been noted that indexing GFR for BSA has limited effects on GFR results in a population of “normal” body size, although the consequences of adjustment can be substantial in extremes of body weight [32]. The absence of associations between BMI categories and BSA-adjusted GFR in our and other studies raises questions regarding the appropriateness of indexing GFR for BSA in overweight populations. In fact, recent papers have demonstrated that obesity is associated with increased GFR, ERPF, and filtration fraction in non-diabetic individuals [4,30,33,34].

We chose to use the Gehan and George formula for BSA estimation, since the Pearson correlation coefficient between this equation and the guideline-recommended Haycock equation was r = 1.0 (P <0.001), with excellent Bland-Altman agreement between the two formulae. This is consistent with previous studies in which the performance of these two equations was very similar. Conversely, in obese patients, the DuBois and DuBois equation underestimated BSA by 3% in males and 5% in females [34].

When measuring GFR from ^51^Cr-EDTA clearance through slope-intercept techniques, sampling is restricted to the second phase of clearance, and systematic errors introduced into the GFR values thus derived must be corrected. Most commonly, correction of the slope-intercept value for the approximation has been done through use of the Bröchner-Mortensen [35] or Chantler [13] equations. The linear Chantler equation uses a constant multiplicative correction factor to adjust GFR values, whereas the Bröchner-Mortensen equation uses a quadratic correction and is dependent on the subject’s BSA [11]. Fleming has compared the two equations and pointed out their limitations: the Bröchner-Mortensen equation underestimated GFR at higher values, with a 10% underestimation at 180 mL/min/1.73 m^2^, while the Chantler equation gave a systematic overestimate of GFR, with the error increasing with GFR, for a 30% overestimate at 180 mL/min/1.73 m^2^[36]. In short, the difference between the two equations is negligible and the Chantler correction can be used accurately.

One strength of the present study was the sample composed entirely of healthy individuals. This stands in contrast to the majority of previous studies, which mostly included potential kidney donors, who are often related by blood to patients with chronic kidney disease. Furthermore, our methods strictly followed the recommendations of the CLSI Guidelines for determining reference intervals.

## Conclusion

We conclude that age, but not sex, must be taken into account in the determination of reference values for glomerular filtration rate. The age-adjusted reference intervals reported in the present study may be reliably adopted to evaluate kidney function.

## Competing interests

The authors declare that they have no competing interests.

## Authors’ contributions

AAS – participated in conception and design, data collection, analysis and interpretation, and drafting of the manuscript. ABP - participated in data collection. LSW, FVV, MJA - participated in data collection and revision of the manuscript. SPS - participated in data collection, conception and design, analysis and interpretation of data, drafting and revision of the manuscript. All authors read and approved the final manuscript.

## Pre-publication history

The pre-publication history for this paper can be accessed here:

http://www.biomedcentral.com/1471-2369/14/54/prepub
